# Supplementary material for: Evaluation of psychological stress in scientific researchers during the 2019–2020 COVID-19 outbreak in China
Source: PeerJ. 2020 Jul 6;8:e9497. doi: 10.7717/peerj.9497 (PMC7346863; doi:10.7717/peerj.9497)
Supplement: Supplemental Information 1 [file peerj-08-9497-s001.docx]

### **Evaluation of psychological stress in scientific researchers during the COVID-19 outbreak in China**

1.Gender

Male

Female

2.Age

18-24

25-39

40-59

≥60

3.Category of school or institution

985、211 universities

General college

University affiliated hospital

Non-university affiliated hospital

Independent research institutes (including research institutes)

Others ()

4. Educational background

Undergraduates

Master candidate (non-graduate year)

Master candidate (graduate year)

PhD candidate (non-graduate year)

PhD candidate (graduate year)

Basic research staff (including postdoctoral)

Clinical medical staff (including postdoctoral)

5.Title of technical post

Professor (researcher, chief physician)

Associate professor (associate researcher, associate chief physician)

Lecturer (assistant researcher, attending physician)

None

6. Question for masters: are you well prepared for graduation?

Not yet

Inadequate preparation

Well preparation

Complete preparation

7. Question for master:do you have any plans for further study?

Yes

No

8. Districts

Wuhan

Non-Wuhan districts in Hubei provinces

Non-Hubei provinces

9. Do you participate in academic research recently?(including students’ programme)

Yes

No

10. What is your study field?

Life-science

Non-life science（engineering, natural science, etc.）

The humanities and social sciences

Others

11.Question for choosing “Life-science” in question 10: what is your programme type?

Public health

Clinical medicine

Basic medicine

Others

12. Question for choosing “Non-life science” in question 10: what is your programme type?

Applied research

Developmental research

Fundamental research/theoretical research

Others

13-36

|  | strongly disagree | disagree | neutral | agree | strongly agree |
| --- | --- | --- | --- | --- | --- |
| want to cry |  |  |  |  |  |
| feel lonely |  |  |  |  |  |
| feel depressed |  |  |  |  |  |
| feel worried. |  |  |  |  |  |
| I think I might go crazy. |  |  |  |  |  |
| Fidgeting and not knowing what to do. |  |  |  |  |  |
| I feel frightened. |  |  |  |  |  |
| I feel nervous and uneasy in the heart. |  |  |  |  |  |
| easy to feel stressful |  |  |  |  |  |
| feel confused |  |  |  |  |  |
| easy to feel anger |  |  |  |  |  |
| feel confused by the pain of the neck or the head |  |  |  |  |  |
| easy to be tired |  |  |  |  |  |
| Can't fall asleep in 30 minutes. |  |  |  |  |  |
| Get up at night |  |  |  |  |  |
| go to the bathroom at the midnight. |  |  |  |  |  |
| It's hard to stay awake when waking up. |  |  |  |  |  |
| feel hopeless |  |  |  |  |  |
| feel harder to concentrate |  |  |  |  |  |
| I don't think I can succeed even if I try hard. |  |  |  |  |  |
| have no interests in the things I used interested in |  |  |  |  |  |
| eat less |  |  |  |  |  |
| be lazy |  |  |  |  |  |
| I've been smoking or drinking a lot lately. |  |  |  |  |  |

37. How’s the project schedule?

Basically, at a standstill

Still under way but at a slower pace than before

Completely unaffected

38. What’s the main reasons for the projects-delaying?

Some researchers (including students) cannot attend

Difficult to obtain (experimental materials/samples) on time

Laboratory facilities are closed or equipment are unavailable

39.What do you feel about the obstruction of research projects you participated in during the COVID-19 outbreak？

Feeling stress and worry

Feeling about to collapse

Few influences to my emotion

Feeling relaxed and happy

40-53

|  | yes | no |
| --- | --- | --- |
| The NCP delays the completion date of scientific research project. |  |  |
| The blocked research projects caused by the COVID-19 has influenced the graduation/project conclusion/funds applications |  |  |
| Original research programmes need to be changed or cut |  |  |
| Epidemic resistance reduces the value of research |  |  |
| The COVID-19 has caused the failures of the original experimental results |  |  |
| The COVID-19 asked more energy input |  |  |
| The COVID-19 has already, or is about to, caused a great loss for projects |  |  |
| The COVID-19 has influenced the original academic exchange activities |  |  |
| The COVID-19 has Influenced the cooperation with other organizations |  |  |
| Colleagues have carried out research projects on NCP |  |  |
| Launch projects concerning the NCP |  |  |
| Pressure on colleagues to carry out research projects on NCP |  |  |
| The block of the scientific researched has caused the adverse effects on your career |  |  |
| The block of the scientific researched has caused the adverse effects on your salaries |  |  |

54. In the following choices, which are beneficial for releasing your pressure? [multiple choice]

1. Prolonging the graduation/project conclusion/the deadline of the application of the funds.

2. Providing funds for the loss of the researchers.

3. Improving the welfare of the researchers

4. Providing free consulting and psychotherapies

5. Assigning professionals to guide projects

6. Encouragement from the superiors

7. Prioritizing the return of researches to work

8. Organizing more academic meetings

9. Develop more academic cooperative projects

10.Others
